# Full-length transcriptome analysis of the bloom-forming dinoflagellate *Akashiwo sanguinea* by single-molecule real-time sequencing

**DOI:** 10.3389/fmicb.2022.993914

**Published:** 2022-10-17

**Authors:** Tiantian Chen, Yun Liu, Shuqun Song, Jie Bai, Caiwen Li

**Affiliations:** ^1^College of Environmental Science and Engineering, Ocean University of China, Qingdao, China; ^2^Key Laboratory of Marine Environment and Ecology, Ocean University of China, Qingdao, China; ^3^CAS Key Laboratory of Marine Ecology and Environmental Sciences, Institute of Oceanology, Chinese Academy of Sciences, Qingdao, China; ^4^Laboratory of Marine Ecology and Environmental Science, Qingdao National Laboratory for Marine Science and Technology, Qingdao, China

**Keywords:** *Akashiwo sanguinea*, harmful algal blooms, full-length transcript, single molecule real-time sequencing, reference resource

## Abstract

The dinoflagellate *Akashiwo sanguinea* is a harmful algal species and commonly observed in estuarine and coastal waters around the world. Harmful algal blooms (HABs) caused by this species lead to serious environmental impacts in the coastal waters of China since 1998 followed by huge economic losses. However, the full-length transcriptome information of *A. sanguinea* is still not fully explored, which hampers basic genetic and functional studies. Herein, single-molecule real-time (SMRT) sequencing technology was performed to characterize the full-length transcript in *A. sanguinea*. Totally, 83.03 Gb SMRT sequencing clean reads were generated, 983,960 circular consensus sequences (CCS) with average lengths of 3,061 bp were obtained, and 81.71% (804,016) of CCS were full-length non-chimeric reads (FLNC). Furthermore, 26,461 contigs were obtained after being corrected with Illumina library sequencing, with 20,037 (75.72%) successfully annotated in the five public databases. A total of 13,441 long non-coding RNA (lncRNA) transcripts, 3,137 alternative splicing (AS) events, 514 putative transcription factors (TFs) members from 23 TF families, and 4,397 simple sequence repeats (SSRs) were predicted, respectively. Our findings provided a sizable insights into gene sequence characteristics of *A. sanguinea*, which can be used as a reference sequence resource for *A. sanguinea* draft genome annotation, and will contribute to further molecular biology research on this harmful bloom algae.

## Introduction

Harmful algal blooms (HABs) have occurred intensively and frequently during the last two decades, and turned into the major marine ecological disaster in coastal waters around the world (Hallegraeff, 1993; Du et al., 2011; Anderson et al., 2012; Chen et al., 2015; Glibert and Burford, 2017). *Akashiwo sanguinea*, a harmful and conspicuous species, is a bloom-forming dinoflagellate capable of discoloring seawater with cells densities exceeding 10^5^ cells L^−1^ (Lu and Hodgkiss, 2004; Kudela et al., 2005; Chen et al., 2019). Annual blooms of *A. sanguinea* have been observed along the coastal waters of China since 1998, resulting in huge economic losses to aquaculture and tourism (Wu et al., 2001; Hao et al., 2011; Chen et al., 2019). Although *A. sanguinea* is not toxigenic, large scale blooms of this species still have a severe impact on the marine ecosystem, which could form surfactant foams under the interaction of wind mixing and surfaction, and cause mass stranding and subsequent mortality of fishes, scallops and birds (Horner et al., 1997; Jessup et al., 2009).

Previous studies mainly focus on the growth, diversity, life cycle, stress responses, physiological and biochemical characteristics of *A. sanguinea* (Matsubara et al., 2007; Chen et al., 2015; Deng et al., 2015; Liu et al., 2015, 2019; Tang and Gobler, 2015; Luo et al., 2017). Furthermore, more efforts have been made to explore the eco-physiology of *A. sanguinea* at the molecular level. For instance, Deng et al. (2015) and Liu et al. (2020) characterized the Hsp 70 gene and the photosynthesis-related genes in *A. sanguinea* during the formation of resting cysts, respectively. The abundance of transcriptome data can facilitate the investigation of biochemical and physiological processes, while only hundreds of Expressed Sequence Tag (EST) sequences and several second-generation transcriptome sequencing data are obtained in NCBI database for *A. sanguinea*, and no full-length transcriptome is openly available, which limited the basic genetic and functional studies in *A. sanguinea*. To date, it is difficult to obtain the reference genome of *A. Sanguinea* through assembly and annotation due to its enormous genome, and transcript sequencing has proved to be one of the most effective technologies for obtaining reliable gene sequences (Erdner and Anderson, 2006; Wisecaver and Hackett, 2010).

The next-generation high-throughput sequencing, also known as second generation sequencing, has been employed to analyze gene expression levels for several marine dinoflagellates, largely increasing the transcript information of these dinoflagellates (Bender et al., 2014; Krueger et al., 2015; Shikata et al., 2019; Li et al., 2021). However, the inherent limitation for the next-generation sequencing is short-read RNA sequencing, which cannot provide a full-length transcript (Wang et al., 2022). Recently, the single-molecule real-time (SMRT) sequencing technology from Pacific Biosciences (PacBio), also called the third generation sequencing, has been proved to be an efficient approach to capture full length sequencing gradually (Wisecaver and Hackett, 2010; Haile et al., 2021; Yang et al., 2021). The full-length cDNA sequences can be generated without assembly *via* PacBio’s SMRT sequencing, dramatically increasing accuracy of alternative splice detection and genes discovery. Even though it is higher error rate (up to 15%) and relevant lower throughput may miss some rare transcript isoforms, these shortcomings can be corrected with high-accurate and high-throughput short reads and/or self-correct *via* circular-consensus reads (Au et al., 2013; Li et al., 2014).

Herein, the marine dinoflagellate *A. sanguinea* was collected and isolated from Jiaozhou Bay, China, and successfully established as continuous culture in June 2020. A full-length transcriptomic analysis of *A. sanguinea* under different nutrition conditions was performed using SMRT sequencing. Based on the obtained transcriptome data, transcript functional annotation, simple sequence repeat analysis, and coding sequence prediction were analyzed. Our findings provided the full-length sequences of *A. sanguinea*, which will be benefit for the further research on the bloom-forming dinoflagellate.

## Materials and methods

### Algal isolation and maintenance

*Akashiwo sanguinea* was obtained and isolated from Jiaozhou Bay, China (36°24′N, 120°11′E) in June 2020. The clonal culture of *A. sanguinea* was established by pipetting single cells under an inverted microscope (Olympus IX71, Japan) to 24-well polystyrene cell culture plates containing sterile f/2-Si medium in natural seawater base (salinity of 30 ± 0.1; Guillard and Ryther, 1962; Kim et al., 2004). Cultures were grown and maintained in an incubator (20 ± 1°C; 12 h:12 h light: dark cycle), with cool white fluorescent light providing 78.14 μE m^−2^ s^−1^. To inhibit the growth of fungus and bacteria, an antibiotic-antimycotic solution, with final concentrations of 0.05 μg ml^−1^ amphotericin B, 100 μg ml^−1^ streptomycin, and 100 I. U. penicillin (Solarbio Inc., Beijing, China), was added to the medium prior to inoculation. This antibiotic mixture had no negative effects on the growth and survival of *A. sanguinea*, as determined in preliminary experiments (Chen et al., 2015; Liu et al., 2020). The stock culture was maintained in the exponential growth phase by transferring into fresh f/2-Si medium bi-weekly.

### Sample processing, RNA isolation, quantification, and qualification

For experiments, stock *A. sanguinea* in exponential growth was inoculated into various types of nutrients: f/2-Si, f/2-Si-N, f/2-Si-P, f/2-Si-NP, natural seawater (as detailed in Table 1). All treatments were conducted in 1 l Pyrex culture flasks containing 800 ml f/2-Si medium (salinity of 30 ± 0.1), with an initial cell density of 2 ± 0.1 × 10^3^ cells ml^−1^. To monitor growth of *A. sanguinea*, algal cell counts were performed by light microscopy every day as described in Chen et al. (2015). For the f/2-Si treatment, 200 ml were concentrated by centrifugation (800 *g*, 5 min) and frozen in liquid nitrogen during the three different developmental stages (exponential growth phase, stationary phase, and decline phase). Other stressed cultures were incubated for 5 days (exponential growth phase) before cells were collected as described above.

**Table 1:** Laboratory setting of different nutritional conditions for growth of *Akashiwo sanguinea*.

Medium type	NO_3_^−^ addition (μM)	PO_4_^3−^ addition (μM)
f/2-Si	883	36.3
f/2-Si-N	–	36.3
f/2-Si-P	883	–
f/2-Si-NP	–	–
Natural seawater	–	–

Total RNA from each culture was extracted using the RNeasy Plus Mini Kit (Qiagen, Valencia, CA, United States), and further treated with RNase-free DNase I (TakaRa, Japan) to remove contaminated genomic DNA. Agilent 2100 Bioanalyzer (Agilent Technologies, Palo Alto, CA, United States) and agarose gel electrophoresis were used to determine the RNA integrity. The purity and concentration of RNA samples were ascertained with the Nanodrop microspectrophotometer (Thermo Fisher Scientific, United States) and Qubit 2.0 fluorometer (Life Technologies, Carlsbad, CA, United States), respectively. Then, RNA samples with a 260/280 ratio of ≥1.8, 260/230 ≥ 1.8 and RIN ≥ 7 were used to construct the Pacbio sequencing library. Finally, equal amounts of RNA samples from different culture conditions were pooled for the following library construction and sequencing.

### Library construction and single-molecule real-time sequencing

A total of 5 μg of total RNA (equally mixed with all RNAs) was used to prepare SMRT libraries. Then, mRNA was reverse-transcribed into cDNA using the Clontech SMARTer PCR cDNA Synthesis Kit (Clontech, CA, United States) according to the Isoform Sequencing protocol. The PCR reactions were optimized to determine the optimal number of amplification cycles for the downstream large-scale PCR procedures. The large-scale double-strand cDNA was produced with the determined number of cycles using Phusion DNA polymerase (NEM, Beverly, MA, United States). The cDNA molecules >5 kb in length were selected using a Blue Pippin™ Size-Selection System (Sage Science, Beverly, MA, United States) and mixed equally with non-size-selected cDNA. Then, another large-scale PCR was preformed, and the amplified and size selected cDNA products were made into SMRTbelll Template libraries. The quality of the libraries was evaluated using the Agilent Bioanalyzer 2100 system. Finally, sequencing reactions were conducted on a PacBio Bioscience Sequel platform (Novogene Bioinformatics Technology Co., Ltd., Beijing, China).

### Data processing

The raw sequencing data of the cDNA libraries was initially processed following the SMRT Link (v 9.0.0) pipeline with parameters: minReadScore = 0.75, minlength = 200. First, high-quality circular consensus sequences (CCSs, HiFi reads) were generated from subread BAM files using the CCS function with parameter settings as: min length = 200, min passes =1, max drop fraction = 0.8, min zscore = −9,999, no polish = TRUE, max length = 15,000, and min predicted accuracy = 0.8. To obtain the full-length nonchimeric (FLNC) reads, the primers, barcodes, polyA tails, and concatemers of full passes were removed. Then, consensus isoforms were identified using the algorithm of ICE (Iterative Clustering for Error Correction) from FLNC and were further polished with non-full length reads to obtain high-quality isoforms with post-correction accuracy above 99% using Quiver (parameters: bin by primer = false, hq quiver min accuracy = 0.99, qv trim 3p = 30, qv trim 5p = 100, and bin size kb = 1). The Cluster Database at High Identity with Tolerance (CD-HIT) program (v 4.6.7) was used to further correct the consensus sequences with the following parameters: −c = 0.99, −G = 0, −T = 6, −AL = 100, −aL = 0.90, −AS = 30, and −aS = 0.99, and the BUSCO (v3.0.2) was used to benchmark transcriptome completeness (Waterhouse et al., 2018). All the raw sequence data have been uploaded to NCBI with the Sequence Read Archive (SRA) number PRJNA827604.

### Functional annotation

Corrected isoforms were searched against Nr (non-redundant protein sequences), Nt (non-redundant nucleotide sequences), Swiss-Prot (a manually annotated and reviewed protein sequence database), KOG/COG (Cluster of Orthologous Groups of proteins), and KEGG (Kyoto Encyclopedia of Genes and Genomes) with BLAST software (v 2.2.26) under a threshold *E*-value ≤ 10^−5^. KEGG pathway analyses were determined using the KEGG Automatic Annotation Server (KAAS1) and HMMER software (Eddy, 1998) was used to search Pfam database (Protein family http://pfam.xfam.org/). Gene Ontology (GO) annotations were performed based on the best BLASTX hit from the NR database using the Blast2GO software (v 2.3.5, -value ≤10^−5^).

### Gene structure prediction

**Figure 1:**
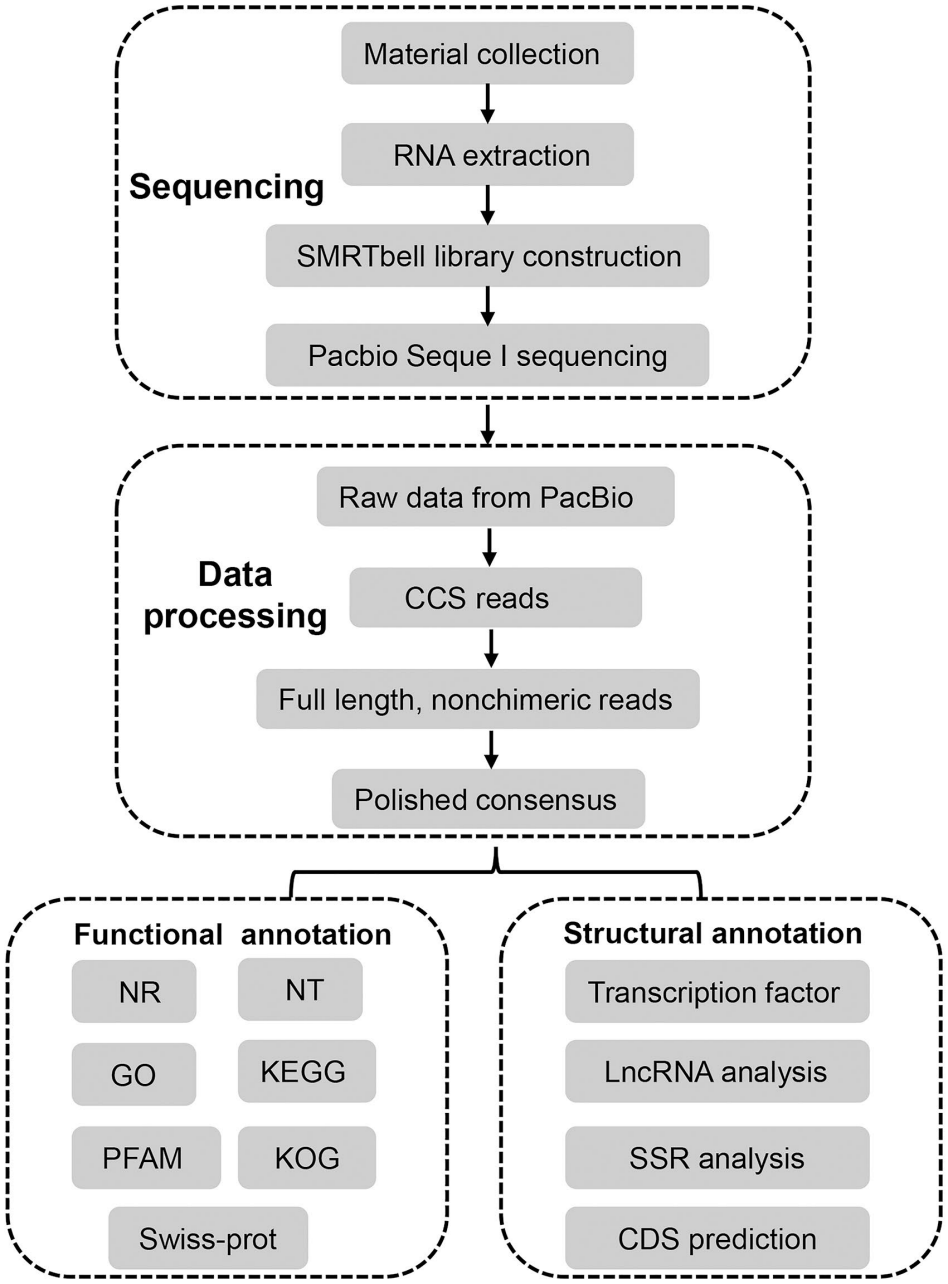
Overall experimental workflow and transcriptome bioinformatics analysis pipeline in this study.

The unigenes were blastx searched against the databases with the *E*-value ≤ 10^−5^ to retrieve a protein sequence for each unigene from either of the four databases in the order of NR, Swiss-Prot, KEGG and KOG, which then located the CDS of the unigene. The unigenes, failing to retrieve a protein sequence, were subjected to ANGEL for CDS prediction (Shimizu et al., 2006). Four tools, including coding potential calculator (CPC), coding-non-coding index (CNCI), coding potential assessment tool (CPAT), and predictor of long non-coding RNAs and messenger RNAs based on an improved *k*-mer scheme (PLEK), were combined to identify LncRNA candidates from putative protein coding RNAs. LncRNAs with >200 nucleotides were selected. Then, the transcripts with encoding ORFs longer than 100 amino acids predicted by these tools were filtered out, and those without coding potential were selected as candidates of lncRNAs. BLASTN was used to get rid of the previously discovered lncRNAs under a criteria of e-value ≤ 1e−10, min-identity = 90% and min-coverage = 85%. Hmmscan against the Plant TFdb database was used to perform TF analysis (Tian et al., 2020). The alternative splicing (AS) events of the transcript isoforms were identified using the Coding GENome teconstruction Tool (Cogent, v 3.3) with the default parameters to divide the transcripts into gene families based on *k*-mer similarity and to reconstruct each family into a coding reference genome based on a De Bruijn graph (Alamancos et al., 2015; Li et al., 2017). The AS events were detected using SUPPA with references. The microsatellite identification tool (MISA) was used to identify simple sequence repeats (SSRs) within the FL transcriptome according to the criteria blow: length-minimum number of repetitions = 2–6 or 3–5 or 4–4 or 5–4 or 6–4 and interruptions of 100 bp (Beier et al., 2017). To characterize full-length transcripts in *A. sanguinea*, an experimental workflow and analysis pipeline was followed as illustrated in Figure 1.

## Results

### Data summary

Based on the PacBio SMRT Sequencing technology, a total of 85.19 Gb of nucleotide data with the average read length of 77,216 bp was obtained. After removing shorter reads (<50 bp in length) and adaptors, a total of 29,370,228 reads (83.03 Gb of nucleotides) were obtained, with an average length and N50 of 2,827 and 3,051 bp, respectively. After merging transcripts with at least two full passes, 983,960 circular consensus sequences (CCSs) with an average length of 3,061 bp were retained. The full-length non-chimeric (FLNC) sequences of the CCSs were further clustered and polished, and 110,200 high-quality (HQ) isoforms were produced, with an average length and N50 of 2,764 and 2,936 bp, respectively. The HQ isoforms were clustered to 26,461 unigenes after removing the sequence redundancy (Table 2).

**Table 2:** Summary of the *A. sanguinea* transcriptome statistics.

Statistical data	*Akashiwo sanguinea*	
Raw reads	Subread number	29,370,228
	Average length (bp)	2,827
	N50 length (bp)	2,003
CCSs	Number of reads	983,960
	Number of CCS bases	3,011,901,560
	CCS read average length (bp)	3,061
	Average number of passes	8
Clustered reads	Number of polished isoforms	110,200
	Polished isoform average length (bp)	2,764
	Polished isoform N50 length (bp)	2,936
Unigenes	Total number	26,461
	Total length (bp)	72,946,143
	Maximum length (bp)	11,908
	Minimum length (bp)	283
	Average length (bp)	2,757
	N50 length (bp)	2,926

### Gene annotations and taxonomy

**Figure 2:**
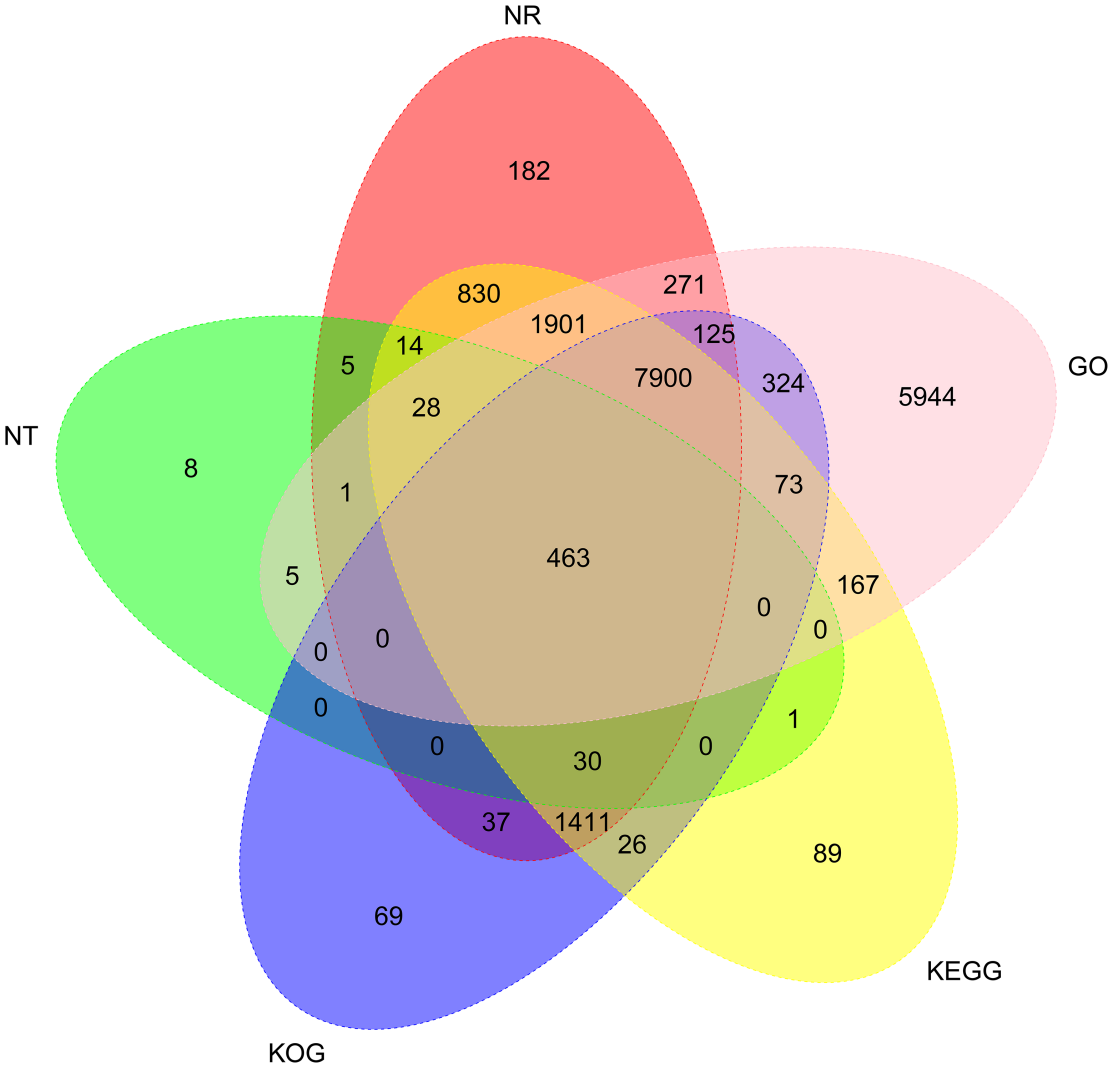
Venn diagram of NR, NT, GO, KOG, and KEGG annotation of the *Akashiwo sanguinea* full-length transcriptome.

To analyze the function of the 26,461 unigenes, five databases, including the non-redundant protein (Nr) database and the NCBI non-redundant nucleotide (Nt) database, the Kyoto Encyclopedia of Genes, and Genomes (KEGG) database, Gene Ontology (GO) database, and Clusters of eukaryotic Ortholog Groups (KOG) database, were used to perform functional annotations. Totally, 20,037 unigenes (75.72%) were annotated prediction and functional annotation of the coded transcripts showed that 13,198 (49.88%), 10,458 (39.52%), 12,933 (48.88%), 12,208 (46.14%), and 17,202 (65.01%) were annotated in the Nr, KOG, KEGG, Nt, and GO database, respectively (Figure 2).

**Figure 3:**
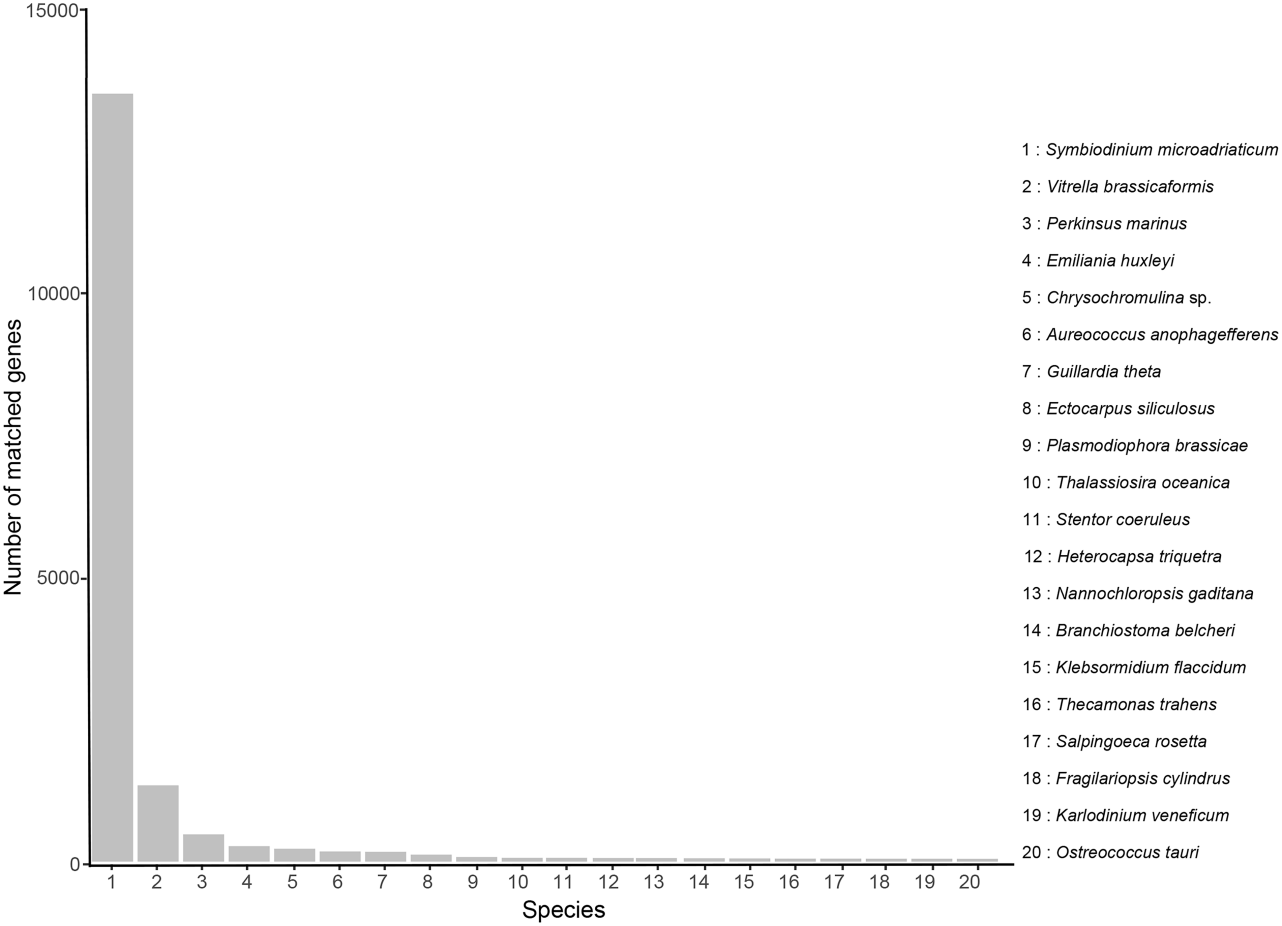
Nr classification of high-quality transcripts of the *A. sanguinea* full-length transcriptome.

With regard to Nr annotation, the top 10 species classifications were *Symbiodinium microadriaticum* (a symbiotic dinoflagellate, 13,725, 51.87%), *Vitrella brassicaformis* (a photosynthetic alveolates, 1,362, 5.15%), *Perkinsus marinus* (a protozoan parasite, 487, 1.84%), *Emiliania huxleyi* (a coccolithophore, 276, 1.04%), *Chrysochromulina* sp. (230, 0.87%), *Aureococcus anophagefferens* (a heterokont alga, 180, 0.68%), *Guillardia theta* (a cryptophyte alga, 175, 0.66%), *Ectocarpus siliculosus* (a filamentous brown alga, 124, 0.47%), *Plasmodiophora brassicae* (a fungus, 82, 0.31%), *Thalassiosira oceanica* (a marine diatom, 66, 0.25%; Figure 3).

**Figure 4:**
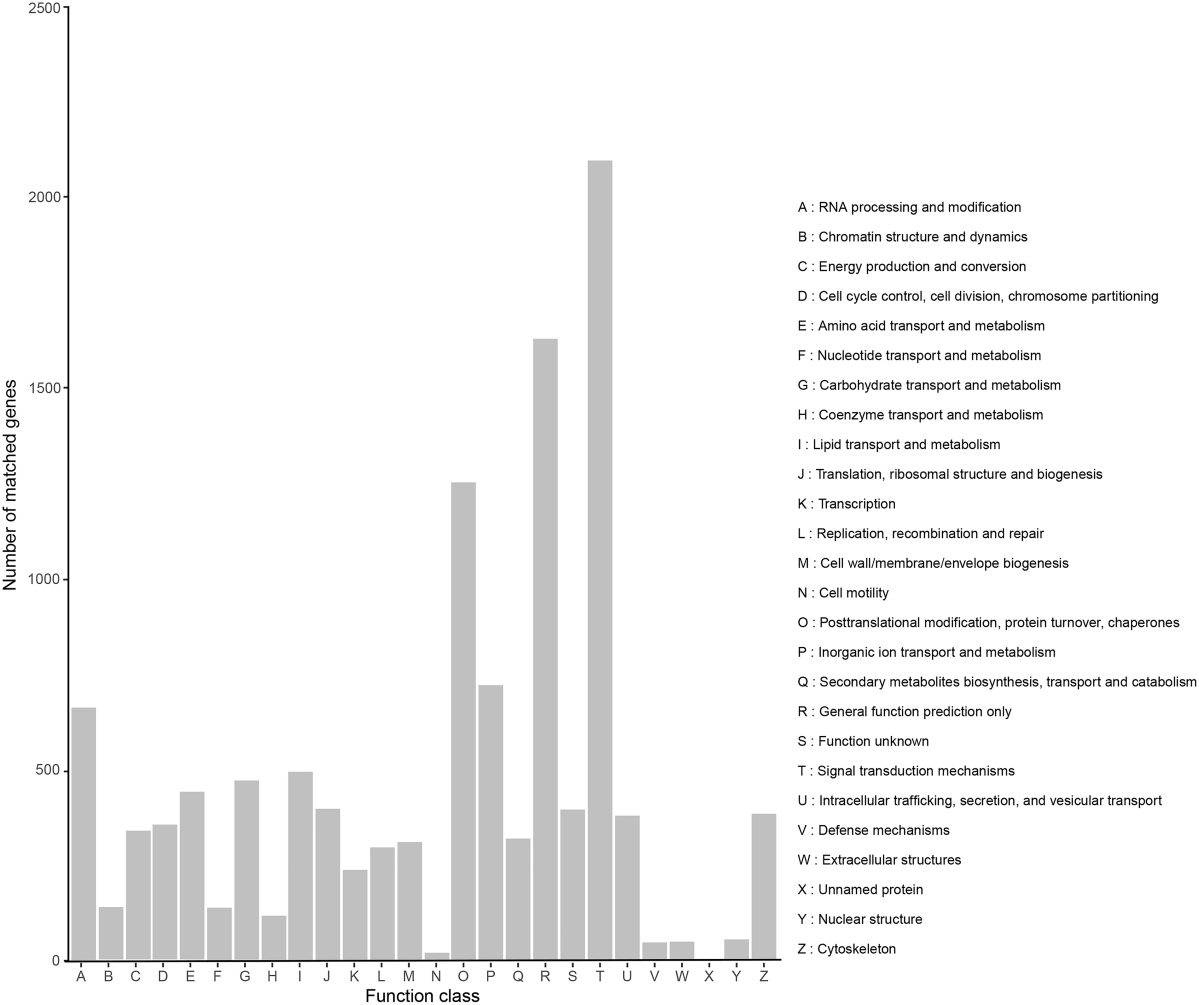
KOG classification of high-quality transcripts of the *Akashiwo sanguinea* full-length transcriptome.

A total of 10,458 transcripts (39.52%) were annotated in the KOG database, which can be assigned to 26 subcategories (Figure 4). The highest percentage of subcategory was the signal transduction mechanisms subcategory, reaching 2,095. The rest were general function prediction only (1,628), posttranslational modification, protein turnover, chaperones (1,252), and cytoskeleton (669).

**Figure 5:**
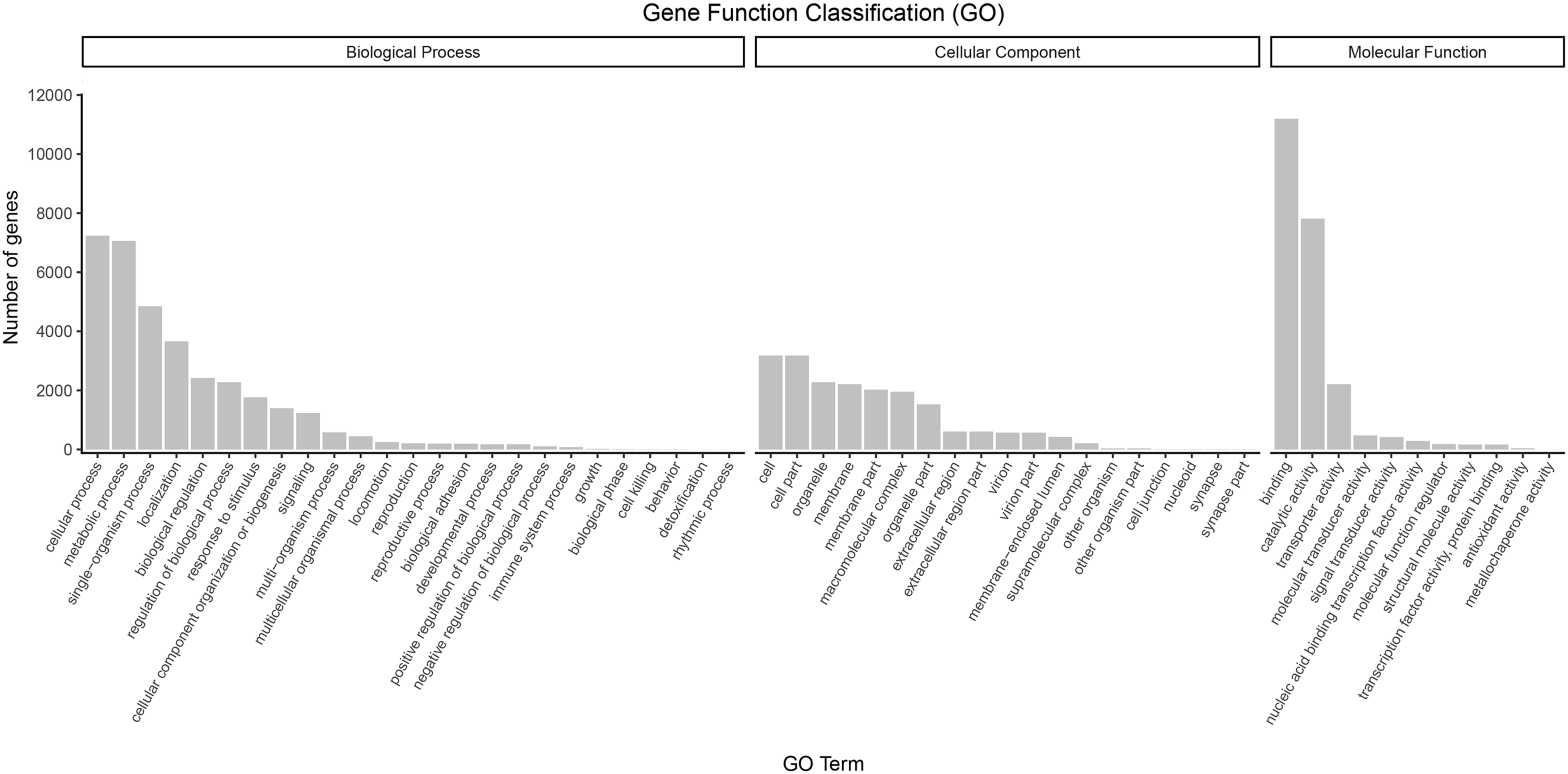
GO classification of high-quality transcripts of the *A. sanguinea* full-length transcriptome.

Regarding functional annotations, 17,202 (65.01%) transcripts were annotated to three major categories of “biological process,” “cellular component,” and “molecular function” in the GO database. The most enriched terms in the biological process category (44.76%) were the “cellular process” (9.43%), “metabolic process” (9.20%), “single-organism process” (6.32%), and “localization” (4.77%) terms. Within the cellular component category (25.33%), the genes involved in “cell” (4.14%), “cell par” (4.14%), “organelle” (2.97%), and “membrane” (2.88%) accounted for the largest proportion. In terms of molecular function (29.91%), “binding” (14.59%), “catalytic activity” (10.18%), “transporter activity” (2.89%), and “molecular transducer activity” (0.62%) covered the most abundant genes (Figure 5).

**Figure 6:**
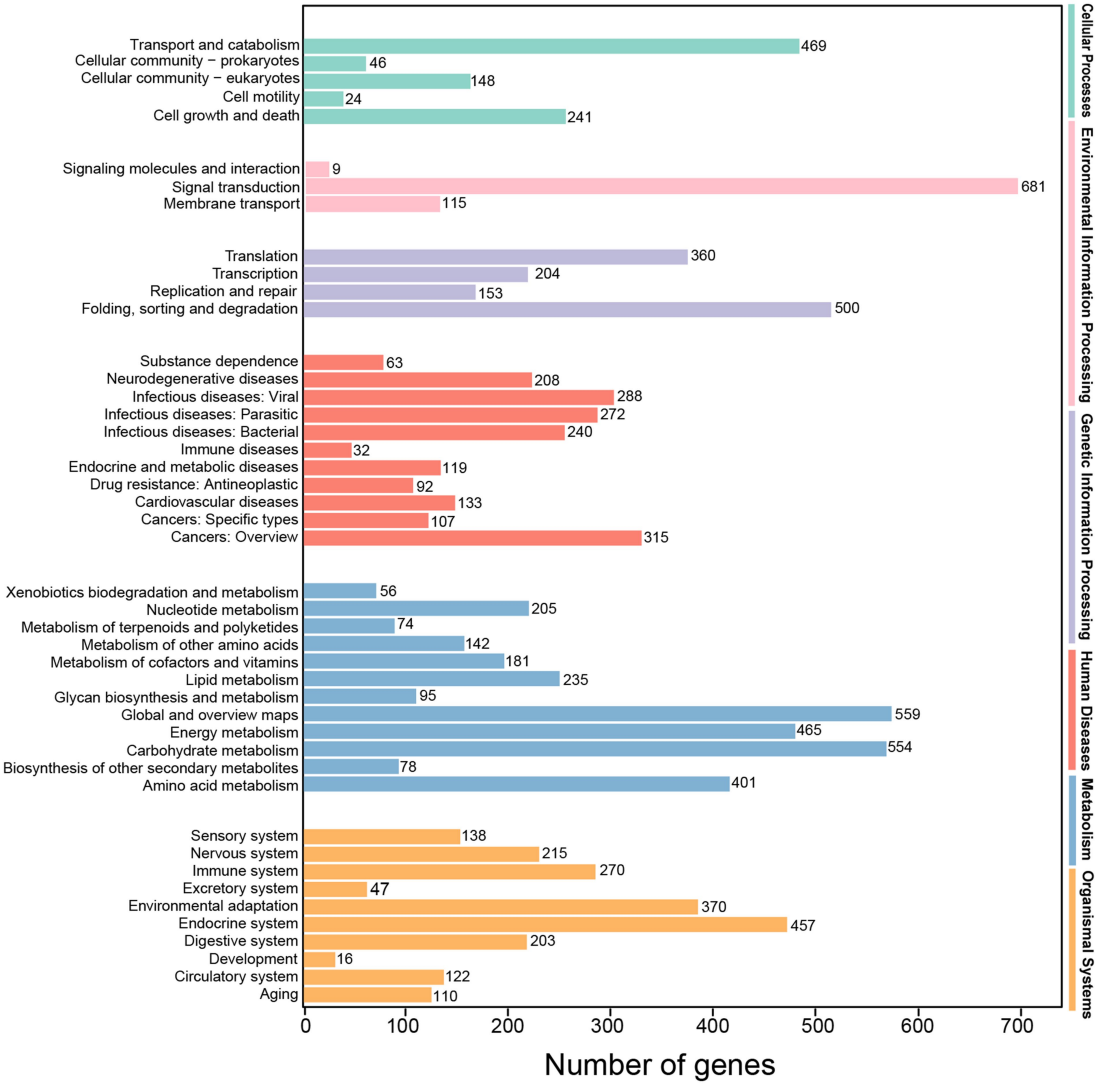
KEGG classification of high-quality transcripts of the *A. sanguinea* full-length transcriptome.

Totally, 13,699 transcripts were classified into the KEGG database, belonging to KEGG’s six primary metabolic pathway (Lev 1) branches, namely, “cellular processes,” “environmental information processing,” “genetic information processing,” “human diseases,” “metabolism” and “organismal systems,” and the most prominent subcategory was “metabolism.” These transcripts were also annotated to 45 secondary pathways (Lev 2) of the six primary metabolic pathway (Lev 1). On the Lev 2 pathway, the genes involved in “signal transduction,” “global and overview maps,” “carbohydrate metabolism” and “folding, sorting and degradation” accounted for the majority (Figure 6).

### Analyses of coding sequence, long non-coding RNAs, and transcription factor

**Figure 7:**
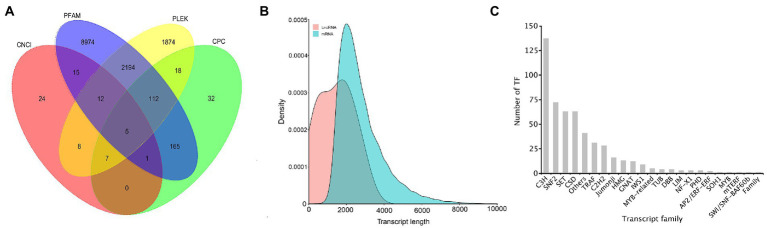
The identification of lncRNAs and the transcript family of the *A. sanguinea* transcriptome. **(A)** The venn diagram of the number of lncRNAs predicted by CPC2, CPAT, PLEK, and CNCI. **(B)** Density and length distributions of mRNAs and lncRNA in *A. sanguinea*. **(C)** Transcript families in the *A. sanguinea* full-length transcriptome.

In 26,461 transcripts with an average length of 2,757 bp, 20,037 (75.72%) CDSs were predicted. Totally, 13,441 lncRNAs (50.80%) were identified using CPC, CNCI, Pfam, and PLEK approaches. The number of putative lncRNAS by CPC, CNCI, Pfam, and PLEK databases were 340, 72, 11,478, and 4,230, respectively. Only 5 common lncRNAs transcripts were predicted in *A*. *sanguinea* by the four methods (Figure 7A). BLASTN was used to get rid of the previously discovered 11 lncRNAs downloaded from ensemble website, and most of lncRNA were identified as novel lncRNAs (Figure 7B). A total number of 514 putative TF members were obtained and categorized into 23 families. The top 10 TFs were ranked according to the number of sequences that were aligned to the transcripts, which were C3H (137), SNF2 (72), SET (63), CSD (63), Others (41), TRAF (31), C2H2 (28), Jumonji (16), HMG (13), and GNAT (12) (Figure 7C).

### Analyses of alternative splicing and simple sequence repeats

**Figure 8:**
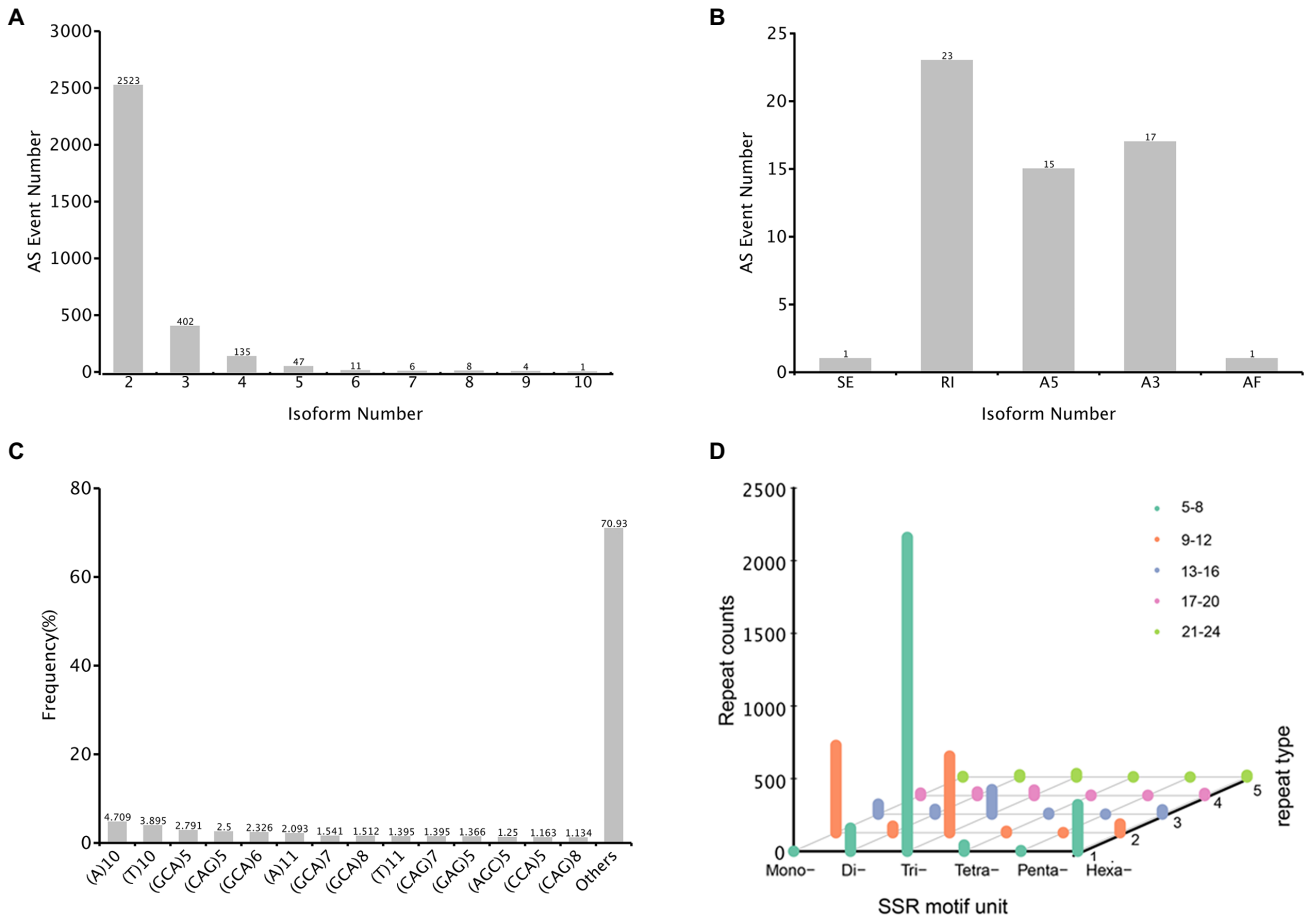
Characterization of structural annotation. **(A)** Distribution of alternative splicing (AS) in each isoform. **(B)** Count of five types of AS. **(C)** The distribution of simple sequence repeats (SSR) type counts. **(D)** SSR motif unit distribution, *x*-axis represented the type of motif unit, *y*-axis represented the count number and *z*-axis represented different repeat number.

The alternative splicing (AS) event provides eukaryotes with peculiarly versatile means of genetic regulation. In total, 3,137 AS events were identified, with the genes containing two isoforms (2,523) ranked the highest, followed by three and four isoforms (Figure 8A). Only 57 events were classified into five AS types, and the major AS types were retained intron (23) and alternative 3′ splice sites (17) (Figure 8B). In the *A. sanguinea* full-length transcriptome, exonskipping, mutually exclusive exons and alternative first exon AS events were not detected.

An simple sequence repeats (SSRs) is a repetitive DNA sequence where certain motifs are repeated. In total, 4,397 SSRs were identified, and these were containing in 32,563 sequences (Figure 8C). The SSR lengths ranged from 10 to 1,354 bp, with a mean of 41.49 bp, and the number of repeat SSR motifs ranged from 5 to 67. Of these SSRs, 2,160 (49.12%) were trinucleotide repeats, most of which consisted of 5–8 repeated sequences; 527 (11.99%) were mononucleotide repeats with 9–12 repeats. In addition, 321 (7.30%) were hexanucleotide repeats with 5–8 repeats (Figure 8D).

## Discussion

*Akashiwo sanguinea* is a commonly observed bloom-forming dinoflagellate distributed worldwide (Trainer et al., 2010; White et al., 2014). Information on partial transcripts of *A. sanguinea* have been obtained by Illumina sequencing in previous studies (NCBI accession: SRR1294461–SRR1294464). While the inherent limitations of Illumina sequencing, including short read length and amplification biases, still limit its application in acquiring a full-length transcript (Abdel-Ghany et al., 2016). Until now, the full-length nucleotide sequence information is still deficient in *A. sanguinea*, which has impeded basic genetic research in this species. With the development of sequencing technologies, PacBio sequencing is widely used in obtaining full-length transcript sequences of microorganisms without assembly (Cheng et al., 2021). Herein, the first high-quality collection of transcripts in *A. sanguinea* was generated by single-molecule long-read PacBio sequencing, and 83.03 Gb clean data were obtained including 983,960 circular consensus sequences (CCSs) and 110,200 high-quality (HQ) isoforms. Long non-coding RNAs (lncRNAs), alternative splicing (AS), simple sequence repeats (SSRs), and transcription factors (TFs) were further revealed in the present study. Our findings provided more accurate annotated unigene information in *A. sanguinea*, which will be useful for the future basic genetic and gene functional studies in this bloom-forming dinoflagellate.

In order to obtain transcripts with very low or no expression in the collected samples, the strategy of pooling of developmental stages and various nutritional conditions has been adopted to capture more complete transcript information (Hoang et al., 2017). The cDNA library was pooled from three developmental stages and five nutritional conditions in the present study. The integrity and reliability of our transcriptome were further confirmed by BUSCO analysis. Higher number of reads was always produced in the Illumina sequencing than that in the PacBio’s SMRT sequencing, however, nearly half of the short contigs generated in former were multiple alignments (Wang et al., 2022). SMRT sequencing provided new insights into capturing long transcript sequences; under normal circumstances, a single read was considered a complete transcript (Sharon et al., 2013; Chao et al., 2018). 97.64% of the generated contigs were >1,000 bp in length in the contigs length distribution of the full-length transcriptome of *A. sanguinea*. Our results demonstrated that SMRT sequencing is an effective and powerful technology for obtaining reliable full-length transcriptome in *A. sanguinea*.

Totally, 20,037 unigenes were annotated in the five public databases, and 6,424 unigenes with unpredicted functions might likely to be species-specific or unknown genes in *A. sanguinea*. With regard to GO annotation, transcripts assigned to categories such as “binding,” “catalytic activity,” “cellular process,” “metabolic process” and “single-organism” were significantly enriched. A total of 10,458 transcripts were assigned to “signal transduction mechanisms subcategory,” “general function prediction only,” and “posttranslational modification, protein turnover, chaperones” according to the KOG annotation analysis. A large of transcripts were involved in specific KEGG pathways, including “signal transduction,” “global and overview maps” and “carbohydrate metabolism.” Additionally, numerous transcripts showed participated in diverse biological pathways and multiple molecular functions. Our results provided a large amount of genetic information for functional investigation in *A. sanguinea*. However, the isoform expression levels was not analyzed in current project, and expression analysis of isoforms derived from one gene in *A. sanguinea* should be analyzed in detail in the future.

LncRNAs emerged as key regulatory molecules in important biological process, including transcription, translation, cellular structure integrity, and sex regulation and aging (Perry and Ulitsky, 2016; Jia et al., 2018). LncRNAs played crucial roles in the nucleus, where they regulate the target genes expression by controlling nuclear architecture and transcription (Wang et al., 2022). LncRNAs also regulated translation, modulated mRNA stability and post-translational modifications in the cytoplasm (Yao et al., 2019). In the current study, 13,441 lncRNAs with a mean length of 2,757 bp were identified. By comparison, the identified lncRNA were much longer than that of known lncRNA (the mean length of 93.09 bp), which showed that SMRT has a better capacity in capturing transcript sequences, especially long transcript sequences.

TFs play a vital role in regulating gene transcription by recognizing and binding specific nucleotide sequences (Fulton et al., 2009). A total number of 514 TFs were obtained and categorized into 23 families, with C3H (137) ranked the highest, followed by SNF2 (72), SET (63), CSD (63), and Others (41). Since all eukaryotic TF families were historically identified and characterized in plants, fungi or animals, these numbers were likely to be underestimated (Rayko et al., 2010). The SNF2 and C3H families were involved in biological processes, such as processing of DNA damage, maintenance of chromosome stability, and RNA processing (Eisen et al., 1995; Delaney et al., 2006), which were commonly present in all the organisms or eukaryote. While the heat shock transcription factor (HSF) family was the two most abundant TF family encoded in diatoms (Rayko et al., 2010). For example, the number of HSF were 187 (51.5%), 70 (33.0%), and 94 (36.4%) in *Thalassiosira weissflogii*, *Phaeodactylum tricornutum*, and *Thalassiosira pseudonana*, respectively (Rayko et al., 2010; Cheng et al., 2021). Besides, Myb and C2H2-type zinc finger TFs were overamplified and constituted the most abundant class of TFs in stramenopile (Rayko et al., 2010).

SSR polymorphic genetic markers, also known as microsatellites, show significant species-specific differences and have been widely used for genetic map construction, functional gene mining, genetic diversity analyses, and molecular marker-related studies (Shen et al., 2014; Feng et al., 2021). A total of 4,397 SSRs identified in *A. sanguinea*, exceeding the SSRs detected in *T. weissflogii* (3,295 SSRs) and *P. tricornutum* (1,390 SSRs) on numbers (Rastogi et al., 2018; Cheng et al., 2021). Herein, the mono-nucleotide (A/T) and tri-nucleotide (GCA/CAG) were the most abundant loci in *A. sanguinea*, and most of SSRs were identified within or around CDS regions and associated with functional genes. The SSRs found here will be of convenience for phylogenetic studies of *A. sanguinea*, and experimental validation should be performed before further using.

Recently, omics analyses have the potential to expand our understanding of the physiological, the initiation and dissipation of algal blooms, and underlying molecular processes of *A. sanguinea*. In the present study, a high-quality and more complete transcriptome analysis of *A. sanguinea* was conducted by the SMRT sequencing, which enabled the generation of full-length transcripts and related analysis, such as efficient gene annotation, lncRNAs, TFs, AS events, and SSRs. Our findings provided a valuable foundation for improving the genome assembly and annotation of *A. sanguinea* by adding accurate genes and structures, which will be helpful to analyze the eco-physiological features of this harmful algae at the molecular level.

## Data availability statement

The data presented in the study are deposited in the NCBI with the Sequence Read Archive (SRA) repository, accession number PRJNA827604.

## Author contributions

TC: conceptualization, investigation, and writing–original draft. YL and SS: methodology, validation, and project administration. SS and JB: methodology and software. CL: supervision, funding acquisition, writing–review and editing, and writing–original draft. All authors contributed to the article and approved the submitted version.

## Funding

This study was financially supported by National Natural Science Foundation of China (grant number 41906122, 41876120, and 41606128), the Key Deployment Project of Centre for Ocean Mega-Science, Chinese Academy of Sciences (grant number COMS2020Q06), and the Marine S & T Fund of Shandong Province for Pilot National Laboratory for Marine Science and Technology (Qingdao) (grant number 2021QNLM040001).

## Conflict of interest

The authors declare that the research was conducted in the absence of any commercial or financial relationships that could be construed as a potential conflict of interest.

The handling editor ZH declared a past collaboration with the author TC.

## Publisher’s note

All claims expressed in this article are solely those of the authors and do not necessarily represent those of their affiliated organizations, or those of the publisher, the editors and the reviewers. Any product that may be evaluated in this article, or claim that may be made by its manufacturer, is not guaranteed or endorsed by the publisher.
